# Development and use of miRNA-derived SSR markers for the study of genetic diversity, population structure, and characterization of genotypes for breeding heat tolerant wheat varieties

**DOI:** 10.1371/journal.pone.0231063

**Published:** 2021-02-04

**Authors:** Sandhya Tyagi, Anuj Kumar, Tinku Gautam, Renu Pandey, Sachin Rustgi, Reyazul Rouf Mir

**Affiliations:** 1 Division of Genetics and Plant Breeding, Faculty of Agriculture (FoA), SKUAST–Kashmir, Wadura, Sopore, Kashmir, India; 2 Division of Plant Physiology, ICAR-Indian Agricultural Research Institute, New Delhi, India; 3 Advance Centre for Computational & Applied Biotechnology, Uttarakhand Council for Biotechnology (UCB), Dehradun, India; 4 Department of Genetics and Plant Breeding, CCS University, Meerut, India; 5 Department of Plant and Environmental Sciences, Clemson University Pee Dee Research and Education Center, Florence, SC, United States of America; Julius Kuhn-Institut, GERMANY

## Abstract

Heat stress is an important abiotic factor that limits wheat production globally, including south-east Asia. The importance of micro (mi) RNAs in gene expression under various biotic and abiotic stresses is well documented. Molecular markers, specifically simple sequence repeats (SSRs), play an important role in the wheat improvement breeding programs. Given the role of miRNAs in heat stress-induced transcriptional regulation and acclimatization, the development of miRNA-derived SSRs would prove useful in studying the allelic diversity at the heat-responsive miRNA-genes in wheat. In the present study, efforts have been made to identify SSRs from 96 wheat heat-responsive miRNA-genes and their characterization using a panel of wheat genotypes with contrasting reactions (tolerance/susceptible) to heat stress. A set of 13 miRNA-derived SSR markers were successfully developed as an outcome. These miRNA-SSRs are located on 11 different common wheat chromosomes (2A, 3A, 3B, 3D, 4D, 5A, 5B, 5D, 6A, 6D, and 7A). Among 13 miRNA-SSRs, seven were polymorphic on a set of 37 selected wheat genotypes. Within these polymorphic SSRs, three makers, namely HT-169j, HT-160a, and HT-160b, were found promising as they could discriminate heat-tolerant and heat-susceptible genotypes. This is the first report of miRNA-SSR development in wheat and their deployment in genetic diversity and population structure studies and characterization of trait-specific germplasm. The study suggests that this new class of molecular makers has great potential in the marker-assisted breeding (MAB) programs targeted at improving heat tolerance and other adaptability or developmental traits in wheat and other crops.

## Introduction

During the last two decades, a variety of ‘Omics’ tools and techniques have been deployed by the crop improvement programs. RNA interference (RNAi) is one of the most important tools often used for genetic manipulation or gene functional characterization. RNAi relies on sequence complementarity for transcriptional suppression and operates through either of the two species of small non-coding RNAs (sncRNAs) dubbed small interfering RNA (siRNA) and microRNA (miRNA) [1]. Among these sncRNAs, miRNAs play a vital role in the gene transcriptional regulation at all the developmental stages and under various biotic/abiotic stresses, including drought and heat [2]. The pace of miRNA discovery has increased over the years due to advances made in the sequencing technology that opened up possibilities of exploring sncRNA populations in staple crops like wheat [1, 3].

Heat stress is one of the most significant abiotic stresses that negatively impact yield and its contributing traits in crop plants [4]. Wheat, a global staple crop, is susceptible to heat stress. Its productivity drops greatly upon exposure to heat stress. Given the rising global temperature, there is an urgent need to explore and utilize novel genetic resources to improve the heat-tolerance of common wheat to sustain yield under projected global warming [5]. The time demands that all possible resources be explored to breed for heat-tolerance, including the untapped repertoire of small RNA molecules such as miRNAs to develop molecular markers or reagents for topical application.

Several QTL studies have been conducted in the past for the exploitation of the genetic variation among the existing cultivars for stress tolerance in different crops [6, 7]. Molecular markers, specifically co-dominant markers like simple sequence repeats (SSRs) flanking these QTLs, play a major role in marker-assisted wheat breeding programs. Generally, SSRs are widely distributed in plant genomes, including coding and non-coding genomic regions [8]. It has been reported earlier that the SSRs present in promoter regions might influence the gene expression level [9] and the ones present in the coding sequences the protein structure and function [9]. Most of the SSRs reported in wheat earlier from the transcribed region belonged to protein-coding sequences [9]. Whereas the non-coding transcribed regions remained underexplored. The importance of non-coding RNAs in plant development and adaptation and the underutilization of this resource caught our attention for the discovery of SSR markers from the non-coding RNA genes.

The availability of the whole genome sequences of the model and non-model plants in the public domain has prompted researchers for the genome-wide identification of miRNA families in plant species, such as rice, maize, and barley [10–13]. In wheat, genome-wide analysis of miRNAs has been reported under heat stress conditions [14, 15]. Recently, SSRs from non-coding RNAs were also developed in wheat [16]. However, to the best of our knowledge, no report is available on the miRNA-derived SSRs (miRNA-SSRs) development in wheat and their use in germplasm characterization for heat stress response. Therefore, in this study, an attempt was made to identify and characterize miRNA-SSRs for heat tolerance in wheat. For this purpose, SSRs were identified from the conserved heat-responsive miRNA genes in wheat and used to characterize a panel of wheat genotypes with contrasting reactions (tolerance/susceptible) to heat stress.

These miRNA-SSRs have potential for use in marker-aided selection (MAS) program for breeding heat-tolerant wheat genotypes. Further, since miRNAs are highly conserved, miRNA-SSRs have likely to be transferable across species.

## Materials and methods

### Plant material and DNA isolation

Thirty-seven diverse wheat genotypes, including 26 heat tolerant and 11 heat susceptible genotypes, were used in this study (S1 Table). These heat tolerant and heat susceptible genotypes are currently being used as national donors and recipients to develop different genetic resources and genetic mapping populations to discover QTLs/genes for heat-tolerance [17–20].

For DNA isolations, leaf samples were collected from one-month-old field-grown plants. DNA isolation was carried out following Saghai-Maroof et al. [21]. After RNAase-treatment, DNA quality was determined by loading the samples on the 0.8% agarose (w/v) gel prepared 1× TAE buffer. DNA quantifications were performed on the Thermo Scientific NanoDrop 1000 Spectrophotometer and dilution were made to the final concentration of 25ng/μl.

### miRNA-SSRs identification and characterization

**Fig 1:**
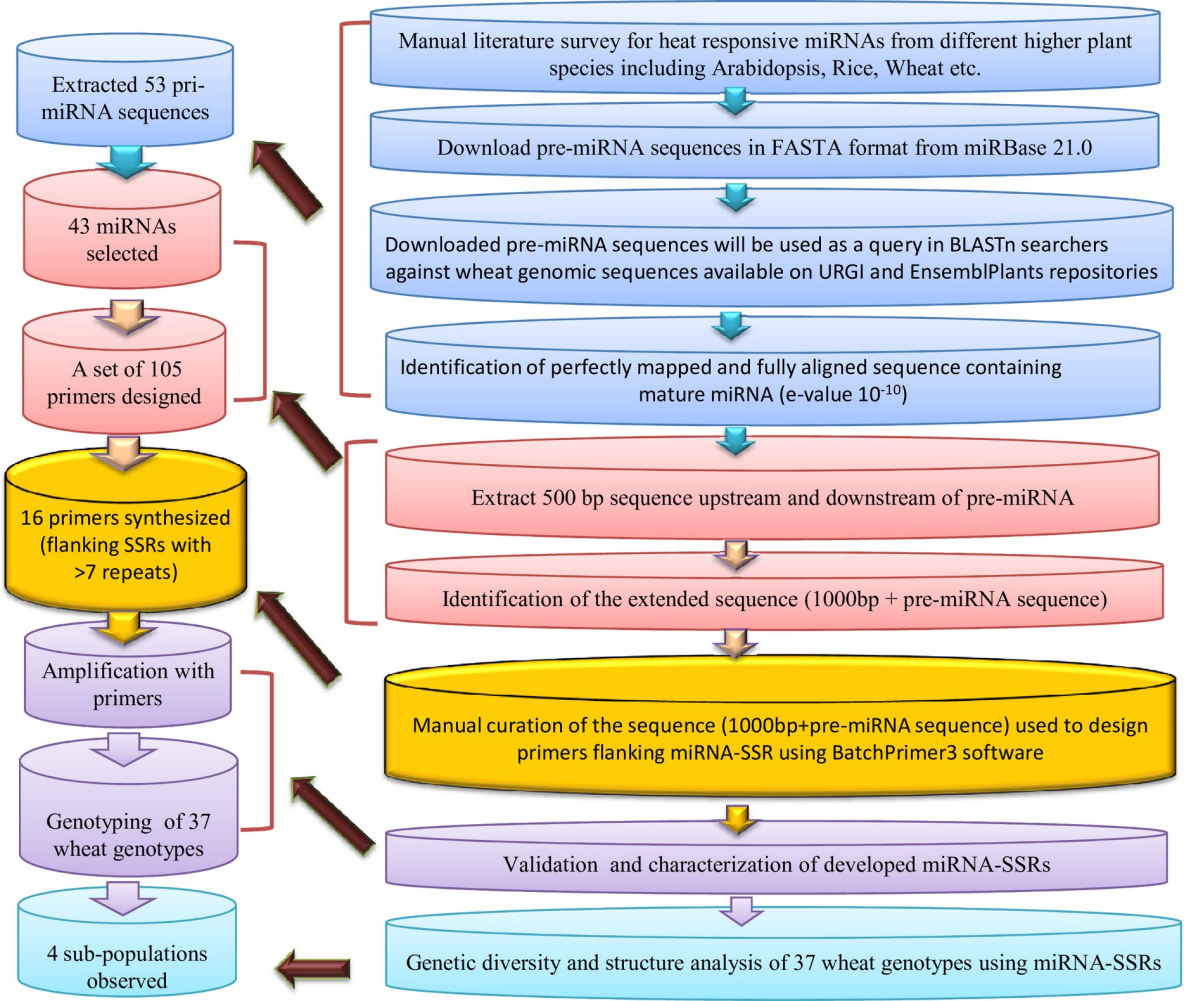
Flow chart showing the pipeline used for the identification and characterisation of miRNA-derived SSRs in common wheat.

An extensive literature-based survey was conducted to identify heat-responsive miRNAs from cereal crops. Reference miRNAs were downloaded from miRBase21.0 (http://www.mirbase.org/) [22], and corresponding full-length transcripts representing precursor (pre)-miRNA were extracted in FASTA format using BioMart tool [23] available on the Ensembl Plants repository [24]. To identify the 500 bp upstream and 500 bp downstream of the pre-miRNA sequence [1000 bp long primary miRNA (pri-miRNA) sequence], BLASTn searches were performed against the latest release of wheat genomic DNA sequence available on EnsemblPlants [24]. The BLASTn searches were performed as described in Mondal et al. [25] and Kumar et al. [26]. In brief, the different steps involved in the process are as follows: i) precursor miRNA sequences of wheat were directly used as ‘query,’ ii) in the case of other plants, corresponding mature miRNAs were taken to find the wheat miRNA orthologue, and then respective pre-miRNA sequences were used as ‘query,’ iii) pre-miRNA sequences of other plants were used directly as a query if mature miRNA orthologue is absent from the wheat miRNA database. As an outcome of BLAST searches, different wheat pri-miRNA sequences of 1000 bp length were identified and scanned for SSRs via Simple Sequence Repeats Identification Tool (SSRIT) [27] (http://archive.gramene.org/db/markers/ssrtoolmarkers/ssrtool) using default settings. Later, the extended miRNA sequences (500 bp upstream + 500 bp downstream) with an SSR motif with ≥ 12 motif length were chosen for designing primers using the online software BatchPrimer3 [28]. A diagrammatic representation of the methodology adopted for miRNA-SSR identification in wheat is presented in Fig 1. The physical chromosomal locations (coordinates) of miRNAs were manually curated from EnsemblPlants following Kumar et al. [29, 30]. The chromosomal locations of the miRNAs were visualized using the ClicO FS tool at a megabase scale [31].

### SSR marker genotyping and data recording

PCR amplification from the genomic DNAs of 37 wheat genotypes with different miRNA-SSR primers was carried out in 20 μl reactions containing 50 ng template DNA, 0.25 μM of each primer, 0.02 mM dNTPs, 1× PCR buffer [10 mM Tris–HCl (pH 8.4), 50 mM KCl, 1.8 mM MgCl_2_, and 0.01 mg/ml gelatine)], and 0.5 U *Taq* DNA polymerase. The PCR reactions were performed on a C1000 Touch™ Thermal Cycler (Bio-Rad Laboratories, Hercules, CA, USA) using the following profile: initial denaturation at 95°C for 5 min followed by 34 cycles of 95°C for 45 sec, 50–52°C for 30 sec (see Table 2 for details), and 72°C for 45 sec. The final extension was performed at 72°C for 7 min. The amplified products were resolved on 10% polyacrylamide gels using a vertical gel electrophoresis unit of Bangalore Genei (glass plate size 19 × 17.5 cm) following Gupta et al. [32]. After electrophoresis, the gels were stained via the silver staining method [33, 34]. The data was recorded as fragment size for different SSR alleles, and the absence of the band was recorded as a null allele.

### Analysis of population structure

A model-based (Bayesian) method was used to evaluate the possible number of sub-populations in a set of 37 common wheat genotypes with miRNA-SSR data. The population structure analysis was performed using the software called STRUCTURE 2.3.4 [35]. Population structure was analyzed by setting the number of sub-populations (K-values) from 1 to 10, and each run was repeated five times. The program was set on 100,000 as burn-in iteration, followed by 150,000 Markov chain Monte Carlo (MCMC) replications after burning along with the admixture model [36]. The STRUCTURE HARVESTER web version v0.6.94 [37] was used to work-out the exact number of sub-populations using a modified delta K (ΔK) method, which provides the real number of clusters [38]. Within a group, genotypes with affiliation probabilities (inferred ancestry) ≥ 80% were assigned to a distinct group, and those with < 80% were treated as admixture, *i*.*e*., these genotypes seem to have mixed ancestry from parents belonging to different gene-pools or geographical origin [39].

### Diversity analyses and population differentiation

A set of 13 miRNA-SSRs was used to genotype 37 wheat genotypes to demonstrate their use in studying genetic diversity. The genotypic data were analyzed using software package GenAlEx version 6.5 [40]. The total number of alleles (Na), the number of effective alleles (Ne), and Shannon’s Information index were determined, and a pair-wise genetic distance matrix was calculated to plot the principal components. Other genetic diversity parameters such as pair-wise Nei’s unbiased genetic distance and genetic identity [41], and gene flow (Nm) were also computed. The Polymorphic Information Content (PIC) for each SSR marker was determined using the software package Power-Marker version 3.25 [42]. The dissimilarity matrix was calculated using DARwin (Dissimilarity Analysis and Representation for WINdows) version 6.0 [43]. The calculated dissimilarity matrix was used for the clustering of 37 genotypes. An un-weighted neighbor-joining (UNJ) method [44] was for dendrogram preparation with 1000 permutations to determine the bootstrap values.

### Analysis of molecular variance (AMOVA) and genetic diversity indices

Analysis of molecular variance (AMOVA) was performed to calculate the level of genetic variation among the genotypes and within the populations. The genetic distance matrix (same as used for PCA) was utilized for conducting AMOVA. The number of sub-populations determined via STRUCTURE was used for AMOVA. The AMOVA was performed using the software program GenAlEx6.5 [40].

## Results

### Discovery and validation of heat-responsive miRNA-SSRs in wheat

Based on the literature survey, we identified 39 miRNA families consisting of 96 members that responded to heat stress in different plant species, including *Arabidopsis*, rice, and wheat. Out of 39 miRNA families, only 14 miRNA families were conserved among all three plant species. These 14 miRNA families comprise 31 members (S2 Table). In wheat, only one (miR156f) out of 31 miRNAs was excluded from further analysis as BLAST searches with it in EnsemblePlants returned high E-value hits. Upon screening for SSRs, two miRNAs (169b and 169c) did not show any SSRs, and five miRNAs (miR156e, miR827, miR399, mir167a, and miR167b) had SSRs with less than seven iterations. The rest of the sequences carried a wide range of repeats that varied from a maximum of six iterations of a tetranucleotide repeat (NNNN)_6_ to a minimum of 27 iterations of a dinucleotide repeat (NN)_27_. A similar strategy was followed in Arabidopsis, where miR159a, miR159b, miR166b, miR168, miR169c, miR169d, miR319, miR398, and miR167b were excluded from analysis due to high E-value returns, miRNA169b carried no repeats, and miRNAs, miR827, miR393a, and miR399 carried SSRs with less than seven iterations. Similarly, in rice, two miRNAs (miR827 and miR167b) were excluded from the analysis due to high E-value returns, three miRNAs (miR827, miR393a, and miR399) carried SSRs with less than seven iterations. Similar to wheat, in rice, miRNA 169b and 169c carried no SSRs. Overall, the dinucleotide repeats were found in high frequency (48), followed in order by tri- (9), tetra- (5), penta- and hexanucleotide (4) repeats (see S2 Table for details).

**Fig 2:**
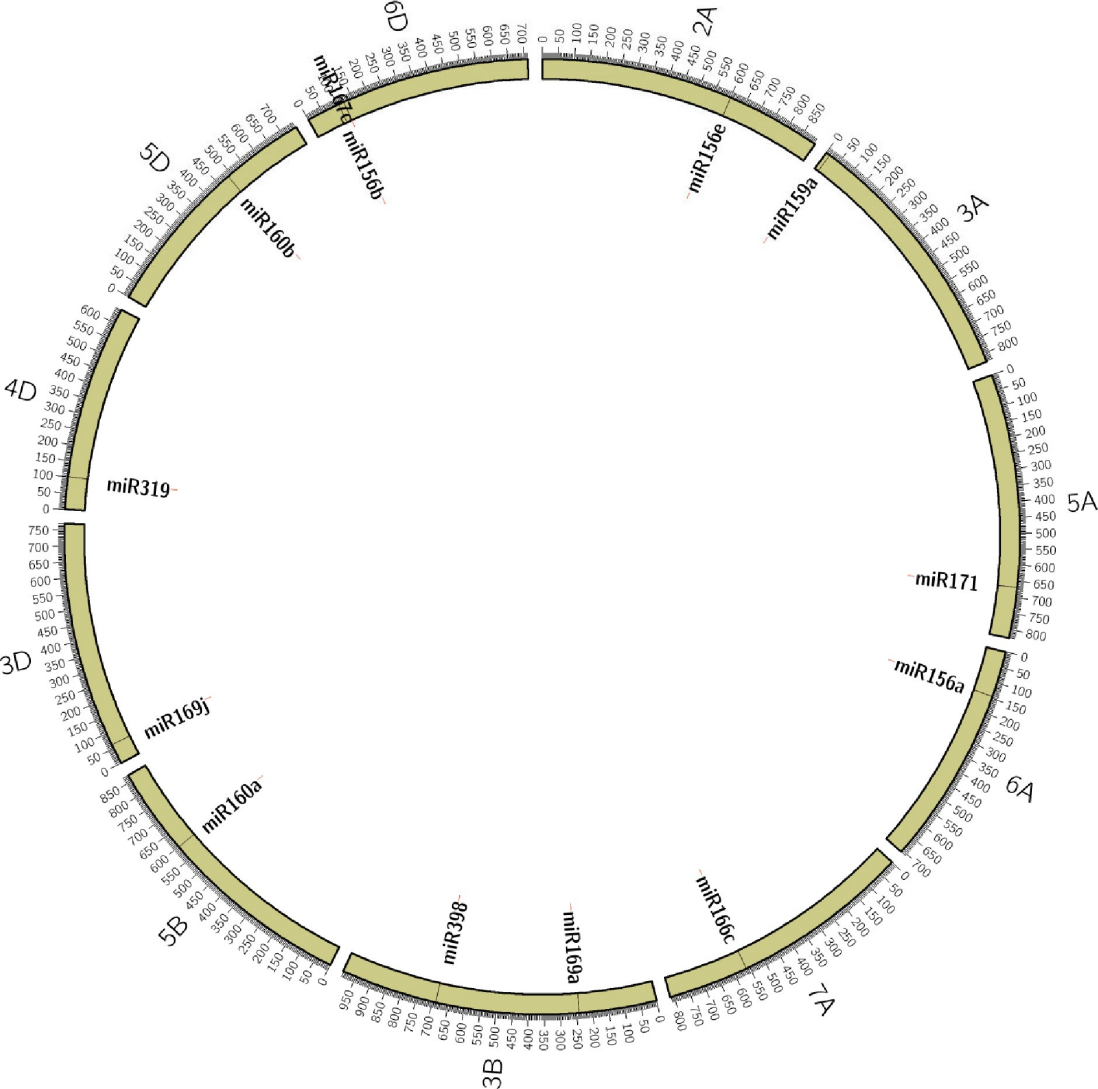
Physical locations of the 13 selected miRNAs on 11 different wheat chromosomes.

For validation, we selected 13 miRNA (Table 1) sequences that carried SSRs with ≥ 12 motif length and targeted them for designing PCR primers flanking the SSRs. The physical locations of the 13 selected miRNAs were determined based on wheat reference genomic DNA sequences available in EnsemblPlants, which were shown in Fig 2. The 13 miRNAs map to 11 different chromosomes (2A, 3A, 3B, 3D, 4D, 5A, 5B, 5D, 6A, 6D, and 7A). Primer pairs for these miRNA-SSRs were designed and tested on a panel of 37 wheat genotypes, including lines with contrasting reactions (tolerance/susceptible) to heat stress. All miRNA-SSR primers amplified products in the expected size range and produced a clear and reproducible banding pattern.

**Table 1:** Details of 13 selected miRNA-SSRs, including gene names, sequences, miRNA lengths, pre-miRNA lengths, and chromosomal locations or genomic coordinates.

Gene names	miRNA Sequences	miRNA lengths	Pre-miRNA lengths	Chromosomal locations	Coordinates
miR156a	TGACAGAAGAGAGTGAGCAC	20	123	Chr 6A	143967938–143968162
miR156b	TGACAGAAGAGAGTGAGCAC	20	183	Chr 6D	104978824-104978846
miR156e	TGACAGAAGAGAGTGAGCAC	20	104	Chr 2A	599784439-599784487
miR159a	TTTGGATTGAAGGGAGCTCTG	21	255	Chr 3A	13739136-13739161
miR160a	TGCCTGGCTCCCTGTATGCCA	21	148	Chr 5B	598031753-598031900
miR160b	TGCCTGGCTCCCTGTATGCCA	21	85	Chr 5D	486487581-486487603
miR166c	TCGGACCAGGCTTCATTCCCC	21	139	Chr 7A	561152178-561152201
miR167c	TGAAGCTGCCAGCATGATCTGC	22	146	Chr 6D	122258436-122258522
miR169a	CAGCCAAGGATGACTTGCCGA	21	226	Chr 3B	242751087-242751111
miR169j	TGAGCCAAGGATGACTTGCCG	21	154	Chr 3D	63154546-63154571
miR171	TGATTGAGCCGTGCCAATATC	21	130	Chr 5A	668149283-668149412
miR319	TGGACTGAAGGGAGCTCCCT	20	211	Chr 4D	102151676-102151886
miR398	TGTGTTCTCAGGTCGCCCCCG	21	120	Chr 3B	3B:690367900-690368017

### Allelic diversity and molecular characterization

**Fig 3:**
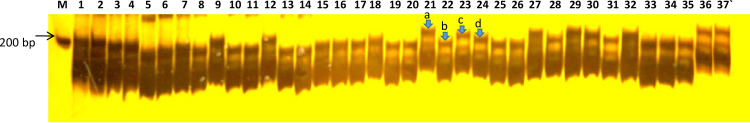
PAGE profile of SSR marker ‘HT-160b’ on 37 wheat genotypes showing length polymorphism. M = 100 base pair ladder, 1–26; heat tolerant wheat genotypes, and 27–37; heat susceptible wheat genotypes. The arrow shows four alleles—a, b, c, and d (marked on genotypes 21–24) amplified by HT-160b.

We tested the use of 13 miRNA-derived SSR markers to characterize 37 wheat genotypes consisting of heat-tolerant and heat-susceptible lines. The analysis of marker data of 13 SSRs led to the identification of 30 alleles. Out of these 13 SSRs, seven showed polymorphism and detected a total of 24 alleles, whereas the remaining six SSRs, namely HT-156a, HT-156b, HT-166c, HT-167c, HT-171, and HT-398, were monomorphic, i.e., detected a single allele each (Table 2). The analysis of PIC indicated that out of the seven polymorphic markers, the highest PIC value (0.375) was observed for miR156e-SSR and the lowest PIC value (0.164) for miR169j-SSR. Markers HT-169j, HT-160a, and HT-160b (Fig 3) were found superior for the analysis of overall genetic diversity as these markers showed more length variation among different genotypes used in the present study and hence considered the best markers for the study of genetic diversity and characterization of wheat germplasm for heat tolerance (Table 2 for a summary).

**Table 2:** Details of 13 miRNA–SSRs, including miRNA gene family, SSR motif, primer sequences, and product size.

S. No	miR gene family /miRNA	Primer Name	SSR Motif	Primer sequences	Tm	Product size
**A**	**miR-156**					
1	miR156a	HT-156a	(CT)_9_	F-TGTACATGGGTTTGATCTTTC	50	153
				R-GTGTGCTCACTCTCTTCTGTC		
2	miR156b	HT-156b	(TGTT)_3_	F-CATCGTTCCTCCTACTAGCTC	52	150
				R-TCGCTCTCTTCTGTCAGC		
3	miR156e	HT-156e	(GCCG)_3_	F-GGTCTTTCCAAACCAAAACTA	50	180
				R-GTGCTCACTCTCTTCTGTCAC		
**B**	**miR159**					
4	miR159a	HT-159a	(TTTC)3	F-AAAGATACGGGCCCTACACC	52	215
				R-GGACCTTGGAGCATTTTCAA		
**C**	**miR160**					
5	miR160a	HT-160a	(TC)_32_	F-GAAGGAAGAGGTGAAAACAAT	50	229
				R-CATGATGCGGTTAGTTTGTAT		
6	miR160b	HT-160b	(TC)_19_	F-GAAGGAAGAGGTGAAAACAAT	50	185
				R-ATTTCCAGGAATCTAAAGCAA		
**D**	**miR166**					
7	miR166c	HT-166c	(GATC)_3_	F-CAGAGAGTAGACGTGCGTAGA	52	173
				R-GAGCAAGATGACCTTAACAGA		
**E**	**miR167**					
8	miR167c	HT-167c	(GGAC)_3_	F-ACTTCTCTCCCTTACCCCTAT	50	167
				R-AGCAAAAATTCCAAGATAACC		
**F**	**miR169**					
9	miR169a	HT-169a	(CAT)_4_	F-GATTTGTCGACCGGATTC	50	149
				R-GCTAAGATGTAGCCAAGAACA		
10	miR169j	HT-169j	(GGA)_7_	F-ACCCAGATTAGGAAAGTCATC	50	143
				R-TATATCCACAGGCAAGTCATC		
**G**	**miR171**					
11	miR171	HT-171	(GCCG)_3_	F-GTGGAATGGTCACTATGATGT	50	127
				R-GCATGAAAGAGCACTGAGATA		
**H**	**miR319**					
12	miR319	HT-319	(GAT)_6_	F-AATTTACAGAGGGGGTACAAA	50	155
				R-AGTGAGCTCCCTCAAACTAAT		
**I**	**mir398**					
13	mir398	HT-398	(ATGC)_3_	F-ATCAACTCATGTGTTCTCAGG	50	150
				R-GCACATTTACCAGTTTGATCT		

### Population structure and genetic relationships

**Fig 4:**
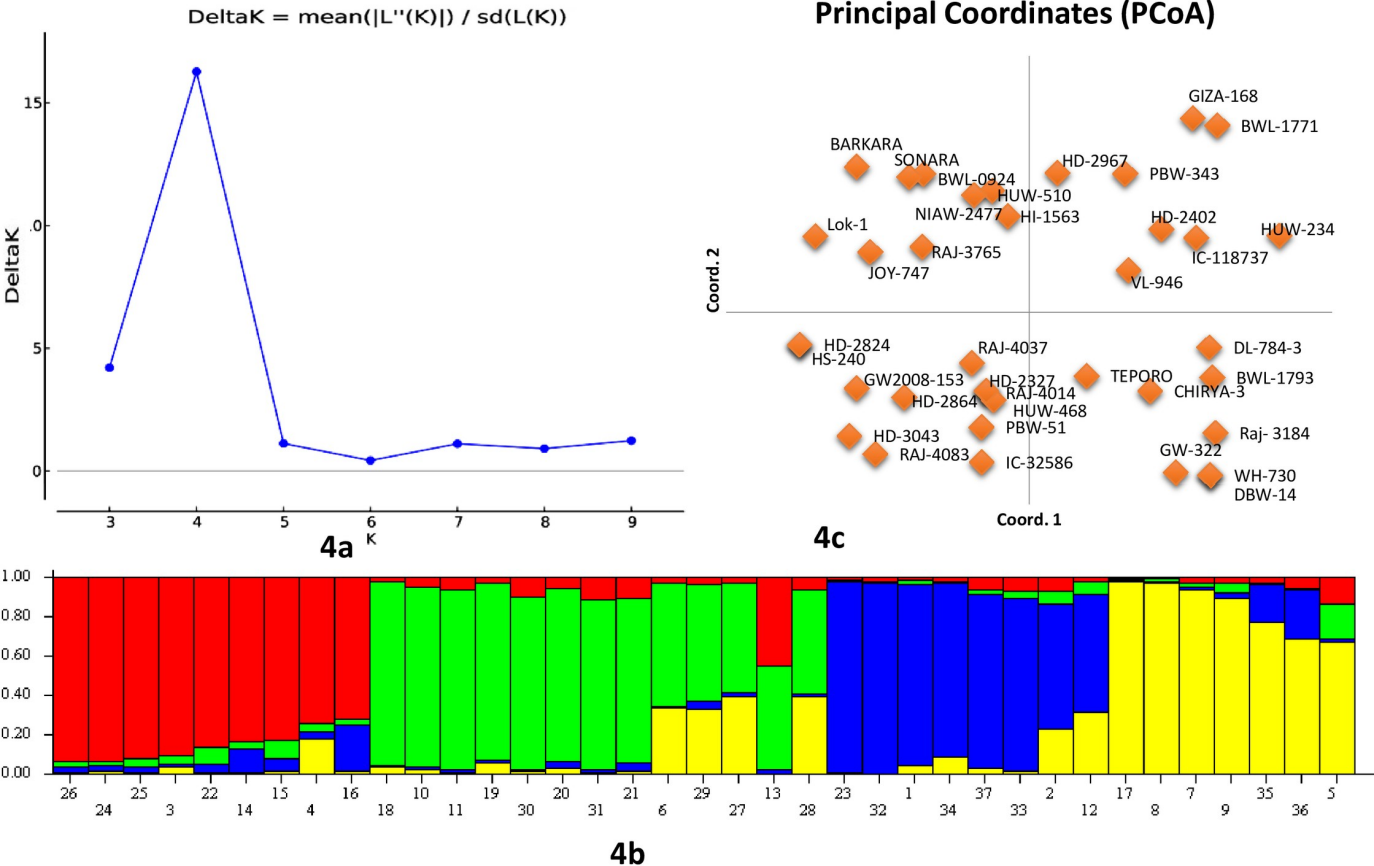
Estimation of the number of groups based on the output from the software STRUCTURE. 4a: Plot of ΔK over K (range 2–10) calculated using the data of 13 miRNA-SSRs genotyped on a set of 37 wheat genotypes. 4b: Bar plot showing the grouping of 37 genotypes into four different clusters. 4c: PCA plot showing the grouping of 37 wheat genotypes into four groups.

For the study of population structure, Bayesian clustering was used in a set of 37 wheat breeding lines/varieties (Fig 4). Genotypic data of seven miRNA-SSRs on 37 wheat genotypes were analyzed using ‘STRUCTURE’ software. The clustering model presumed the existence of sub-populations ‘K clusters’ in the test population, and the Evano test suggested them to be four clusters ‘K = 4’ (Fig 4A and 4B). The analysis of four subpopulations identified by structural analysis indicated that 10, 12, 8, and 7 genotypes fall in four subpopulations, namely P1, P2, P3, and P4, respectively. Similar results were also obtained from PCoA, which clustered 37 wheat genotypes into four clear groups (Fig 4C). A significant genetic divergence was observed among the four subpopulations from each other (Table 3). All 24 polymorphic alleles were recorded in sub-populations P1 and P2, while in sub-populations P3 and P4, only 23 alleles were present. A total of nine genotypes were found to show admixture.

**Table 3:** Analysis of molecular variance (AMOVA) calculated using the data of 13 miRNA-SSRs genotyped on a set of 37 wheat genotypes.

Source	df	SS	MS	Est. Var.	%
**Among Pops**	3	16.518	5.506	0.139	3%
**Within Pops**	33	139.914	4.240	4.240	97%
**Total**	36	156.432		4.379	100%

### Genetic differentiation of populations

The allelic information about four subpopulations identified by STRUCTURE analysis was used in GenAlex 6.5 software for AMOVA analysis and the genetic diversity indices. The AMOVA revealed that 3% of the total variation was found among/between subpopulations, while the rest of the variation (97%) was due to individuals/genotypes present within subpopulations (Table 3). These results demonstrated that genetic differentiation among subpopulations was low and within subpopulations was high. The mean values of different genetic parameters of the four subpopulations were provided in Table 4. Genetic variation of individual loci in 37 wheat genotypes and four populations including the sample size, number of different alleles (Na), the number of effective alleles (Ne), Shannon’s index (I), expected heterozygosity (He), and unbiased expected heterozygosity (uHe) were provided in S3 and S4 Tables, respectively.

**Table 4:** Genetic diversity parameters including the number of different alleles (Na), number of effective alleles (Ne), information index (I), expected heterozygosity (He), and unbiased heterozygosity (UHe) in four sub-population of the 37 genotypes.

Pop	N	Na	Ne	I	He	UHe	%P
**Pop1**	12.000	1.733	1.471	0.409	0.274	0.298	76.67
**Pop2**	10.000	1.467	1.398	0.349	0.234	0.260	63.33
**Pop3**	8.000	1.433	1.424	0.352	0.241	0.275	60.00
**Pop4**	7.000	1.433	1.446	0.359	0.248	0.289	60.00
**Mean**	9.250	1.517	1.435	0.367	0.249	0.281	65.00

N = Sample size; Na = Number of alleles; Ne: Number of effective alleles; I: Information index; He: Expected heterozygosity; UHe: Unbiased Heterozygosity

### Cluster analysis of 37 wheat genotypes

**Fig 5:**
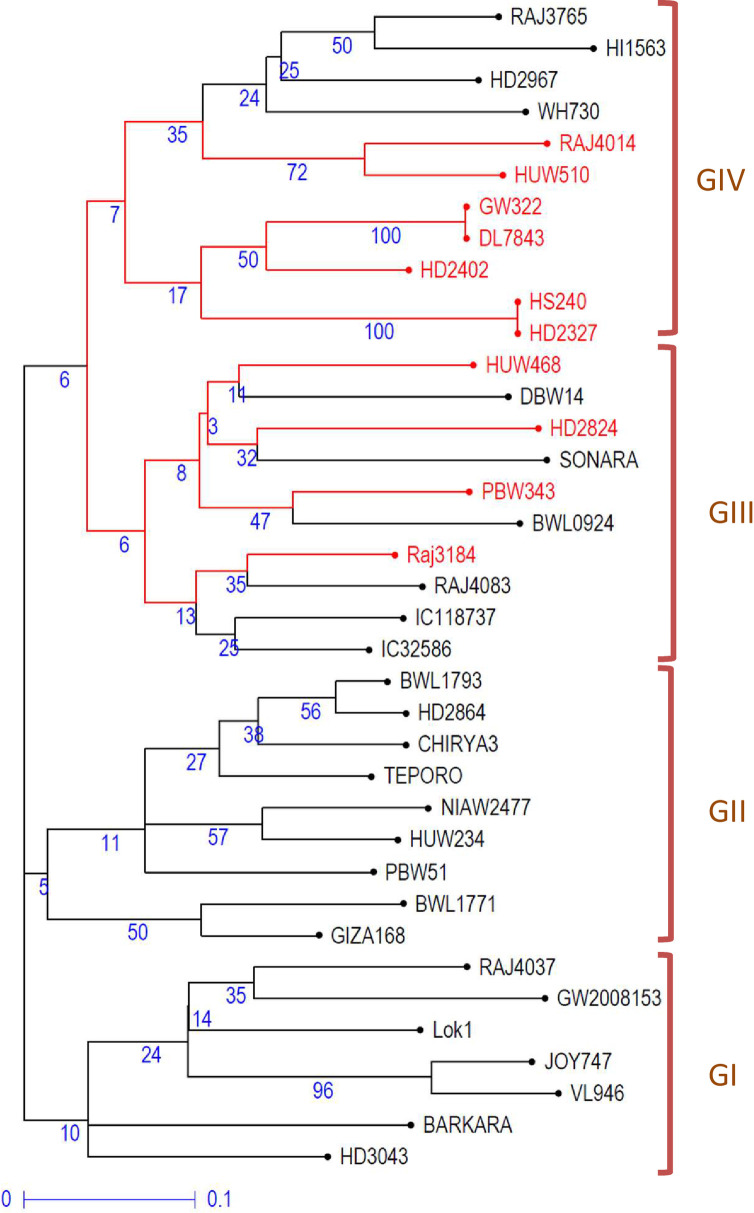
Un-weighted neighbor-joining tree showing the grouping of 37 genotypes based on miRNA-SSRs data. The genotypes highlighted in red color are heat-susceptible, while genotypes written in black color are heat-tolerant. Values given in blue color are bootstrap values.

The data from miRNA-SSRs profiling were also used to study the genetic diversity among the 37 genotypes. The results led to the clustering of 37 genotypes into four main groups/clusters (GI, GII, GIII, and GIV) (Fig 5). There were 7, 9, 10, and 11 genotypes, respectively, in GI, GII, GIII, and GIV. GI and GII included all heat stress-tolerant genotypes, while groups GIII and GIV comprised 6 or 4 and 4 or 7 heat-tolerant and heat-susceptible genotypes, respectively. This clustering of heat-tolerant and heat-susceptible genotypes in a cluster may be because of the existence of the same genetic background/pedigree of some genotypes. For instance, the parental genotypes ‘PBW343’ (heat susceptible) and its derived line ‘Raj4083’ (heat tolerant) clustered together in GIII. Similarly, heat susceptible line ‘HD2402’ is in the pedigree of heat-tolerant genotype ‘Raj3765,’ both clustered in GIV. The Jaccard’s similarity index between the pairs of wheat genotypes ranged from 32% to 89.5%, with a mean similarity index of 55.9%.

## Discussion

Micro RNAs are important small non-coding RNA molecules, which can be differentiated from other RNA classes based on the following characteristics: (i) miRNAs are derived from genomic loci distinct from the protein-coding genes, (ii) miRNAs are processed in two steps from long transcripts, dubbed primary miRNA (pri-miRNA) and precursor miRNA (pre-miRNA), and (iii) miRNAs sequences are conserved among related organisms. Plant miRNAs have been described for the first time in *Arabidopsis thaliana* and later in other species. At present, miRNAs have been reported in dozens of plant species [3, 12, 13, 45], and their sequences have been deposited in a public miRNA database, miRBase (http://www.sanger.ac.uk/cgi-bin/Rfam/mirna/browse.pl). Several miRNAs identified and characterized in crop plants were found to be involved in the development processes, phytohormone signaling, flowering, sex determination, and biotic/abiotic stress responses [45]. Lately, miRNA-based molecular markers have been developed and used for various purposes in crop plants, including genetic diversity analysis [16, 24, 46].

SSRs, among other molecular markers, were extensively used for genetic diversity analysis, QTL mapping, genome-wide association analysis, and marker-assisted breeding [47, 48]. The earlier studies suggested that the SSRs present within the genes (genic SSRs) are more appropriate for molecular breeding than random genomic SSRs [49–51], most of which are derived from the protein-coding or untranslated regions (UTRs) of the plant genomes [52]. The occurrence of SSRs within the non-coding miRNA genes and their role in biotic and abiotic stress adaptation has only recently caught attention [53]. As widely known, miRNAs are 20–22 nucleotide stretches of non-coding RNAs (ncRNAs), which regulate gene expression in plants under various biotic and abiotic stresses [24, 53, 54]. The location of SSRs within pri-miRNAs is uncertain [55]. Also, in pri-miRNAs, SSRs differ in their number, repeat iterations, and motif type (mononucleotide to hexanucleotide repeats) [55].

Heat stress is affecting wheat yields globally. To sustain wheat productivity under the alarming projections of further global warming, there is a need to identify wheat varieties with tolerance to heat stress via a combination of forward and reverse genetic approaches. Recent literature surveys suggested the role of miRNAs in abiotic stress adaptation in plants, including heat stress in wheat [56]. Besides, several studies reported the characterization of heat stress-tolerant wheat germplasm [57, 58] using SSR markers (derived from protein-coding or random genomic regions). However, to the best of our knowledge, none of the earlier studies attempted identification of SSRs from trait-specific miRNA genes and their use in the characterization of wheat genotypes for tolerance to heat stress. However, SSRs within the miRNA precursors have been identified and used for the diversity analysis in rice [24, 59].

*In-silico* studies were performed in the past to identify SSRs from the miRNA genes in *Arabidopsis* [26] and rice [24], but similar studies lacked in wheat. Given the importance of miRNAs in crop improvement, we screened the heat-responsive miRNA genes in wheat for SSRs and used them to characterize wheat genotypes with contrasting heat stress responses. In the present study, 13 selected miRNA-SSRs were found to map on the chromosomes of six homeologous groups 2, 3, 4, 5, 6, and 7. Several QTLs for heat stress response in wheat were earlier identified to localize on these chromosomes [58]. Out of 13 miRNA-SSRs, seven detected polymorphism due to variations in the repeat unit number (length polymorphism). It was repeatedly reported that the mutation rate is high in SSRs with higher repeat numbers, which contributes to the high polymorphism rate at these loci [26].

The sub-populations differed for the number of detected SSR alleles due to the presence of common and unique alleles in different sub-populations. It is tempting to propose that the change in SSR repeat units, responsible for length polymorphism, contributes to transcriptional regulation of heat-responsive genes and resulting heat-induced adaptive change in the tolerant genotypes. All seven polymorphic SSR markers showed the multi-allelic pattern in the 37 wheat genotypes, which indicate a high level of genetic diversity in wheat germplasm selected for mapping the heat response traits and breeding for heat tolerance. As evident from the dendrogram, GI and GII sub-clusters contained all heat-tolerant genotypes, whereas GIII and GIV possessed both heat-tolerant and heat-susceptible genotypes, which may be due to the overlapping genetic backgrounds of the genotypes with heat-tolerance and heat-susceptibility as also evident from their pedigrees (S1 Table) or the insufficient number of markers used for diversity analysis. The clustering of heat-tolerant genotypes in GI and GII sub-clusters showed potential of miRNA-SSRs as diagnostic tools for screening wheat germplasm for heat tolerance after necessary validation using a mapping population. These SSRs might be tested for association with heat-tolerance using different methods, including single marker analysis (SMA).

In earlier studies, miRNA-160 has been reported to target AUXIN RESPONSE FACTOR (ARF) genes that determine the auxin/cytokinin balance in *Arabidopsis*, maize, soybean, rice, and wheat [60–65] and contribute to heat and drought tolerance. Further support for these observations comes from a wheat study where *Ta-miR160* was shown to respond highly to heat stress [66]. Like miRNA-160, miRNA-169 has been shown as a down regulator of the genes under drought stress conditions in maize [62, 67] and *Arabidopsis* [68]. Furthermore, a high throughput sequencing study reported the heat stress-induced expression of *Ta-miR169* and *Ta-miR160* [69]. These earlier studies corroborated our findings that the three miRNA-SSRs (HT-169j, HT-160a, and HT-160b), which discriminated heat-tolerant and heat-susceptible genotypes and clustered them in separate groups, belonged to the two miRNA families that play important roles in heat stress adaptation in different plant species. Hence, the miRNA-SSRs derived from these miRNA genes would serve as the highly informative tool in breeding for heat tolerance in wheat and likely other related crops. Expansion or contraction of the repeats at the SSR loci has been reported to play a major role in the transcriptional regulation of genes. Therefore, this variation in SSR length at miRNA genes might be contributing to changes in gene expression patterns of the downstream genes in response to environmental cues and increasing/decreasing the stress tolerance level of genotypes.

In summary, seven miRNA-SSR markers were found polymorphic, but out of these 7 miRNA-derived SSRs, only three (HT-169j, HT-160a, and HT-160b) that showed association with two miRNA families, miR169 and miR160, were promising. These SSRs largely proved useful in clustering two groups of wheat genotypes with contrasting heat stress responses.

## Conclusion

A set of 13 SSR markers were derived from the heat-responsive miRNA gene in wheat. These SSRs are distributed on 11 different wheat chromosomes, namely 2A, 3A, 3B, 3D, 4D, 5A, 5B, 5D, 6A, 6D, and 7A. When tested on a set of 37 wheat genotypes, seven out of 13 miRNA-SSRs showed polymorphism. Some of these markers (HT-169j, HT-160a, and HT-160b) discriminated between heat-tolerant and heat-susceptible genotypes, hence could serve as diagnostic tools for heat tolerance breeding efforts in the future after necessary validation. These markers have also shown promise in studying genetic diversity and population structure of 37 wheat genotypes with contrasting heat stress responses. The estimates of diversity determined using miRNA-SSR markers should carry more weight since these markers are derived from genes and have more chances of identifying functional variability among genotypes. The diverse genotypes identified during the present study will be used as donors to develop genetic resources for breeding heat-tolerant wheat varieties. As more miRNA genes will be characterized, these miRNA-SSRs will play an important role in serving as sources of highly informative molecular markers, which will aid the marker-assisted breeding (MAB) efforts in different plant species.

## Supporting information

S1 Raw image Uncropped and labelled original image of gel provided as Fig 3 in the original manuscript. Figure panel within the box was used to generate Fig 3 from the original image.
(PDF)

S1 Table Details of 37 heat tolerant/susceptible wheat genotypes used in the study. (PDF)

S2 Table Details of 14 heat responsive miRNA families conserved in wheat, Arabidopsis, and rice. (PDF)

S3 Table Genetic variation of individual locus in 37 wheat genotypes including the sample size, number of different alleles (Na), the number of effective allele (Ne), Shannon’s index (I), expected heterozygosity (He), and unbiased expected heterozygosity (uHe). (PDF)

S4 Table Genetic variation of individual locus in four populations including the sample size, number of different alleles (Na), the number of effective allele (Ne), Shannon’s index (I), expected heterozygosity (He) and unbiased expected heterozygosity (uHe). (PDF)
