# Supplementary material for: Development and use of miRNA-derived SSR markers for the study of genetic diversity, population structure, and characterization of genotypes for breeding heat tolerant wheat varieties
Source: PLoS One. 2021 Feb 4;16(2):e0231063. doi: 10.1371/journal.pone.0231063 (PMC7861453; doi:10.1371/journal.pone.0231063)
Supplement: S1 Raw image — Figure panel within the box was used to generate Fig 3 from the original image. (PDF) [file pone.0231063.s001.pdf]

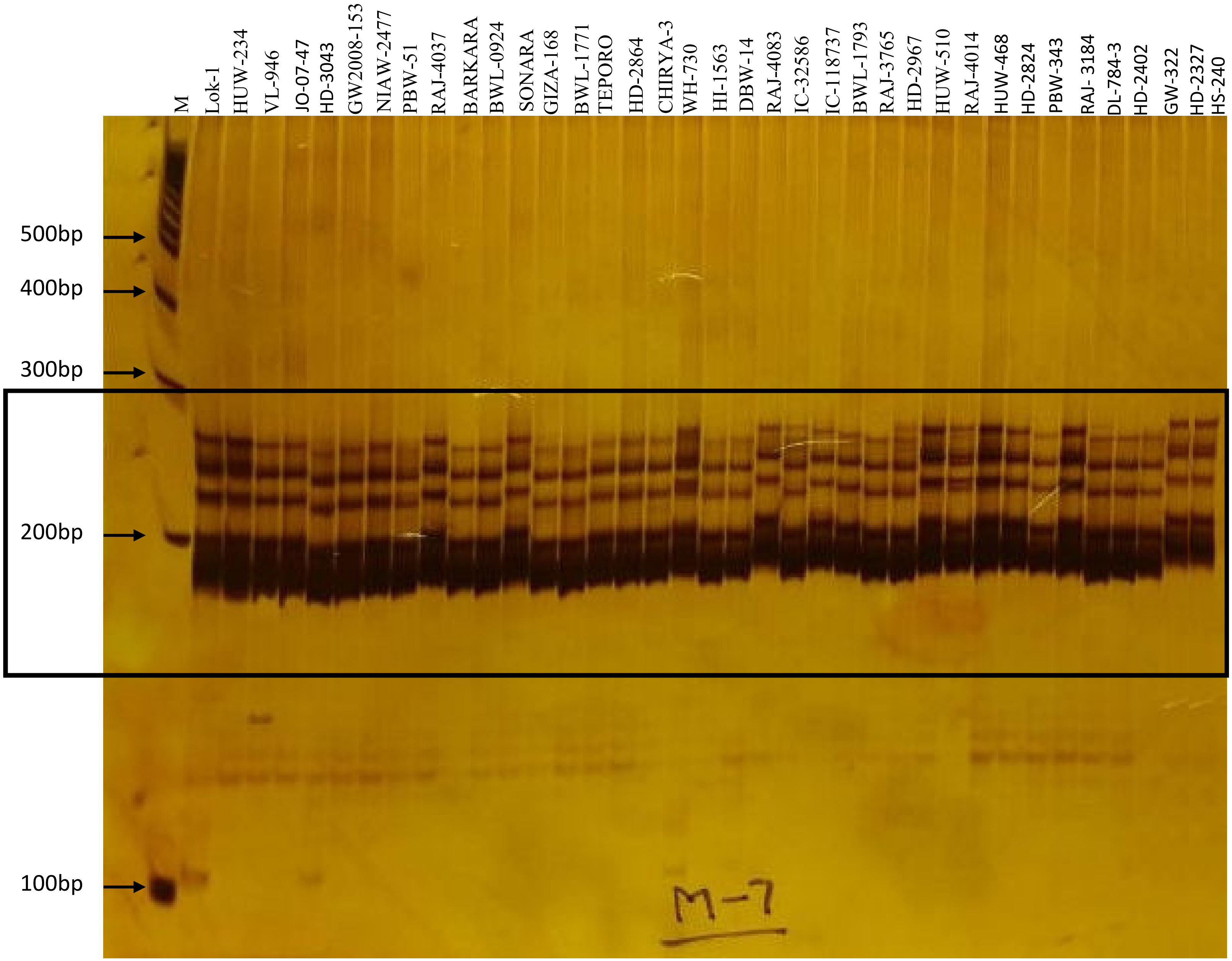

S1 Raw Image: Uncropped and labelled original image of gel provided as Fig 3. in the original manuscript. Figure panel within the box was used to generate Fig 3. from the original image.
