# Supplementary material for: Development and use of miRNA-derived SSR markers for the study of genetic diversity, population structure, and characterization of genotypes for breeding heat tolerant wheat varieties
Source: PLoS One. 2021 Feb 4;16(2):e0231063. doi: 10.1371/journal.pone.0231063 (PMC7861453; doi:10.1371/journal.pone.0231063)
Supplement: S1 Table — (PDF) [file pone.0231063.s002.pdf]

Supplementary Table 1: Details of 37 heat tolerant/susceptible wheat genotypes used in the study.

S. No.	Genotype	Tolerant vs Susceptible	Pedigree	Name of developing centre	Area/zone of Adoption
1	Lok-1	Tolerant	S-308 x S-311	SANOSARA	CZ
2	HUW-234	Tolerant	HUW 12*2 / CPAN 1666//HUW12	-	NEPZ
3	VL-946	Tolerant	HW 2045/VW 2185	IIWBR, Karnal	-
4	JO-07-47	Tolerant	HEAT TOLERANT NURSERY 2015-16	IIWBR, Karnal	-
5	HD-3043	Tolerant	PJN/BOW//OPATA*2/3CROC_1/A. Squarrosa(224) //OPATA	ICAR-IARI, New Delhi	NWPZ-TS,RIR
6	GW2008-153	Tolerant	HEAT TOLERANT NURSERY 2015-16	IIWBR, Karnal	-
7	NIAW-2477	Tolerant	HD2402/VL639	-	NWPZ
8	PBW-51	Tolerant	PBW 51*3/ KS90 H 450/ Moro // HW4444 (HUW234*3/CLr19)	-	-
9	RAJ-4037	Tolerant	DL788-2/RAJ3717	RAU, Durgapur	IR, TS
10	BARKARA	Tolerant	Viginta/ Danubia	-	-
11	BWL-0924	Tolerant	N/A	-	-
12	SONARA	Tolerant	YT54/N10B//2*Y54	New Delhi	NPZ/CZ
13	GIZA-168	Tolerant	Mil/Buc//Seri	Egypt	-
14	BWL-1771	Tolerant	N/A	-	-
15	TEPORO	Tolerant	-	-	-
16	HD-2864	Tolerant	DL 509-2/DL 377-8	New Delhi	CZ
17	CHIRYA-3	Tolerant	-	-	-
18	WH-730	Tolerant	CPAN 2092/Improved Lok I	CCSU, Hisar	-
19	HI-1563	Tolerant	MACS2496*2/MC10	-	-
20	DBW-14	Tolerant	RAJ 3765/PBW 343	DWR	NEPZ
21	RAJ-4083	Tolerant	PBW343/UP2442/WR258/UP2425	SKNAU-RARI, Durgapura	PZ-LS,IR
22	IC-32586	Tolerant	-	-	-
23	IC-118737	Tolerant	-	-	-
24	BWL-1793	Tolerant	ND/VG9144	-	Germplasm line
25	RAJ-3765	Tolerant	HD 2402/VL 639	RAU Durgapur	NEPZ
26	HD-2967	Tolerant	ALD/COC//URES/HD 2160 M/HD 2278	ICAR-IARI, New Delhi	-
27	HUW-510	Susceptible	HD 2278 / HUW 234 // DL 230 - 16	-	PZ
28	RAJ-4014	Susceptible	DL 8025/K 9011	Rajasthan	-
29	HUW-468	Susceptible	CPAN 1962 / TONI // LIRA / PRL	-	NEPZ
30	HD-2824	Susceptible	-	-	NEPZ
31	PBW-343	Susceptible	PBW 343*3/ KS 90 H 450/Moro// HW4444(HUW234*3/CLr19)	-	NWPZ
32	Raj- 3184	Susceptible	-	-	-
33	DL-784-3	Susceptible	KAL*4/TR380.27*4/3AG/3/HD2281	IARI, New Delhi	NEPZ
34	HD-2402	Susceptible	HD2177//CN067/BB/3/HD 2160/4/HD 2236	-	NEPZ
35	GW-322	Susceptible	GW173/GW196	JAU, Junagarh	IR, TS
36	HD-2327	Susceptible	-	-	CZ
37	HS-240	Susceptible	AU /KAL- BB//WOP'S'/P AVON'S'	-	NHZ
