# Supplementary material for: Development and use of miRNA-derived SSR markers for the study of genetic diversity, population structure, and characterization of genotypes for breeding heat tolerant wheat varieties
Source: PLoS One. 2021 Feb 4;16(2):e0231063. doi: 10.1371/journal.pone.0231063 (PMC7861453; doi:10.1371/journal.pone.0231063)
Supplement: S2 Table — (PDF) [file pone.0231063.s003.pdf]

Supplementary Table 2: Details of 14 heat responsive miRNA families conserved in wheat, Arabidopsis, and rice.

S. No.	Known miRNA	Wheat			Arabidopsis			Rice		
		**Chromosome**	**E value**	**Motifs**	**Chromosome**	**E value**	**Motifs**	**Chromosome**	**E value**	**Motifs**
**1**	**miR-156**									
	miR156a	5DL	0.022	$(CT)_9$	5DL	0.0046	$(CT)_9$	3DS	0.000015	$(CT)_8$ $(TG)_7$ $(GC)_7$
	miR156b	5AL	0.022	$(TGTT)_3$	6DS	0.029		5DL	0.007	$(CT)_9$
	miR156c	5DL	4.7E-113	$(CT)_9$, $(CCT)_4$	5DL	0.0038	$(CT)_9$	5DL	0.00037	$(CT)_9$
	miR156d	5DL	0.088	$(CT)_9$	3DS	0.0011	$(CT)_8$ $(TG)_7$ $(GC)_7$	6BS	3.4E-10	NO SSR
	miR156e	6AS	2E-122	<7	3DS	0.000016	$(CT)_8$ $(TG)_7$ $(GC)_7$ $(GGT)_4$	2DL	2.7E-13	$(GCCG)_3$ $(GTAG)_3$ $(AGTGGG)_3$
	miR156f	5DL	5.4	-	3AS	3.5E-10	$(TG)_{43}$ $(GGT)_4$	5DL	0.000031	$(CT)_9$
	miR156g	5DL	0.088	$(CT)_9$	3DS	0.00095	$(CT)_8$ $(TG)_7$ $(GC)_7$ $(GGT)_4$	5DL	0.0000011	$(CT)_9$
**2**	**miR159**									
	miR159a	3AS	0.00068	$(CTC)_4$ $(TTTC)_3$	3AS	1.8	-	3AS	0.00018	$(CTC)_4$ $(TTTC)_3$
	miR159b	3B	2.1E-89	$(TTCT)_3$ $(TAGATG)_4$	3AS	0.12	$(CTC)_4$ $(TTTC)_3$	5DL	1.5E-22	$(TTCT)_3$
**3**	**miR160**	5BL	5.8E-77	$(TG)_6$ $(CT)_6$ $(TC)_{32}$ $(GGA)_4$ $(GTGA)_4$	5DL	0.012	$(TC)_{19}$ $(GAG)_4$ $(GTGA)_3$	6BS	8.7E-13	$(GA)_9$ $(CT)_9$ $(TG)_{10}$ $(TC)_8$
**4**	**miR-166**									
	miR166a	5BL	0.0084	$(ATC)_7$	4BL	0.00043	$(TC)_{48}$ $(AG)_{21}$	1AL	1.6E-12	$(TTTG)_3$
	miR166b	5BL	0.0084	$(ATC)_7$	4DL	2	-	7DL	6E-13	<7
	miR166c	6DL	0.0084	$(CTT)_5$ $(GAGC)_3$	7BL	0.0054	$(GA)_7$ $(TC)_6$ $(GATC)_3$	4AS	1.3E-09	$(AG)_{16}$
	miR166d	4DL	2	$(TC)_8$ $(GA)_{12}$	7BL	0.000068	$(GA)_7$ $(TC)_6$ $(GATC)_3$	1AL	0.000019	$(TTTG)_3$
**5**	**miR168**	6AS	0.022	$(AC)_8$ $(CCCCGC)_4$	4AL	NO HIT FOUND	-	6AS	8.3E-10	$(AC)_8$ $(CCCCGC)_4$
**6**	**miR169**									
	miR169a	3B	0.0084	$(CAT)_4$ $(TCTCAG)_3$	3B	0.0024	$(CAT)_4$ $(TCTCAG)_3$	3B	0.00000046	$(CAT)_4$ $(TCTCAG)_3$
	miR169b	2DL	0.0084	NO SSR	2DL	0.0073	NO SSR	2AL	0.000079	NO SSR
	miR169c	2DL	0.011	NO SSR	-	1.2	-	2DL	3E-13	NO SSR
	miR169d	5BL	0.0084	$(GGA)_7$ $(TAGA)_3$	3B	0.37	$(CAT)_4$ $(TCTCAG)_3$	5AL	0.00095	$(TACG)_3$ $(TCCT)_3$
**7**	**miR172**									
	miR172a	6DL	0.0084	$(CTC)_4$ $(TTC)_7$ $(TTC)_4$ $(GTA)_4$ $(AGAT)_3$ $(GGCC)_3$ $(ATTT)_3$ $(GCGGT)_4$	3B	0.00094	$(CTT)_6$ $(TTC)_5$ $(AGAT)_3$	1DL	0.00000027	$(TC)_{18}$ $(TC)_7$

	miR172b	6DL	0.022	$(CTC)_4$ $(TTC)_7$ $(TTC)_4$ $(GTA)_4$ $(AGAT)_3$ $(GGCC)_3$ $(ATTT)_3$ $(GCGGT)_4$	3B	0.00022	$(CTT)_6$ $(TTC)_5$ $(AGAT)_3$	3AL	0.00000017	$(TCA)_5$ $(CAGC)_3$
**8**	**miR827**	2AL	0.0084	<7	2BS	0.52	<7	2BS	0.52	<7
**9**	**miR171a**	5DL	2.7E-66	$(TGCT)_3$, $(CGCC)_3$, $(GCCG)_3$, $CATG)_4$, $(GGGCA)_4$	1AL	0.0000048	$(GA)_{13}$ $(TC)_{12}$ $(CTAG)_3$ $(CTTCC)_3$	7AL	1.8E-23	<7
**10**	**miR393**									
	miR393a	1BL	0.0084	$(GCAG)_5$	7BL	0.0012	<7	3DL	0.0000015	$(AGC)_4$
	miR393b	2DL	0.0084	$(GAA)_9$ $(TGCT)_3$	2DL	0.0013	$(GAA)_9$ $(TGCT)_3$	1DL	0.0000036	<7
**11**	**miR319**	4DS	2.2E-114	$(GAT)_6$ $(CATG)_3$	4AL	0.11	$(ATG)_4$ $(CATG)_3$	3AS	8.9E-12	<7
**12**	**miR398**	3B	8.8E-57	$(CTCC)_3$ $(ATGC)_3$	5DL	1.1	-			
**13**	**miR399**	1AL	6.5E-64	<7	7BL	0.0012	<7	3DL	0.0000015	$(AGC)_4$
**14**	**miR167**									
	miR167a	5AL	1.4E-27	<7	5BL	0.083	$(CATC)_3$ $(TCCGC)_3$ $(TCCCGC)_3$	5AS	0.000000092	$(TCTG)_3$
	miR167b	5AL	6.5E-23	<7	5BS	0.0000041	$(TCTG)_3$ $(AGTT)_3$	5BL	4.6E-13	$(CATC)_3$ $(TCCGC)_3$ $(TCCCGC)_3$
	miR167c	6DS	4.7E-25	$(CCT)_5$ $(GGAC)_3$	5BL	0.52	$(CATC)_3$ $(TCCGC)_3$ $(TCCCGC)_3$	5DL	4.6E-13	$(TGAGA)_4$
