# Supplementary material for: Development and use of miRNA-derived SSR markers for the study of genetic diversity, population structure, and characterization of genotypes for breeding heat tolerant wheat varieties
Source: PLoS One. 2021 Feb 4;16(2):e0231063. doi: 10.1371/journal.pone.0231063 (PMC7861453; doi:10.1371/journal.pone.0231063)
Supplement: S3 Table — (PDF) [file pone.0231063.s004.pdf]

Supplementary Table 3: Genetic variation of individual locus in 37 wheat genotypes including the sample size, number of different alleles (Na), the number of effective allele (Ne), Shannon's index (I), expected heterozygosity (He), and unbiased expected heterozygosity (uHe).

Locus	Band Freq.	p	q	N	Na	Ne	I	He	uHe
**Locus 1**	0.405	0.229	0.771	37.000	2.000	1.546	0.538	0.353	0.358
**Locus 2**	0.595	0.363	0.637	37.000	2.000	1.861	0.655	0.463	0.469
**Locus 3**	0.270	0.146	0.854	37.000	2.000	1.332	0.415	0.249	0.252
**Locus 4**	0.595	0.363	0.637	37.000	2.000	1.861	0.655	0.463	0.469
**Locus 5**	0.595	0.363	0.637	37.000	2.000	1.861	0.655	0.463	0.469
**Locus 6**	0.135	0.070	0.930	37.000	2.000	1.150	0.254	0.130	0.132
**Locus 7**	0.189	0.100	0.900	37.000	2.000	1.218	0.324	0.179	0.182
**Locus 8**	0.027	0.014	0.986	37.000	2.000	1.028	0.072	0.027	0.027
**Locus 9**	0.243	0.130	0.870	37.000	2.000	1.293	0.387	0.226	0.229
**Locus 10**	0.514	0.303	0.697	37.000	2.000	1.730	0.613	0.422	0.428
**Locus 11**	0.216	0.115	0.885	37.000	2.000	1.255	0.356	0.203	0.206
**Locus 12**	0.027	0.014	0.986	37.000	2.000	1.028	0.072	0.027	0.027
**Locus 13**	0.216	0.115	0.885	37.000	2.000	1.255	0.356	0.203	0.206
**Locus 14**	0.297	0.162	0.838	37.000	2.000	1.372	0.443	0.271	0.275
**Locus 15**	0.189	0.100	0.900	37.000	2.000	1.218	0.324	0.179	0.182
**Locus 16**	0.297	0.162	0.838	37.000	2.000	1.372	0.443	0.271	0.275
**Locus 17**	0.595	0.363	0.637	37.000	2.000	1.861	0.655	0.463	0.469
**Locus 18**	0.324	0.178	0.822	37.000	2.000	1.414	0.468	0.293	0.297
**Locus 19**	0.676	0.431	0.569	37.000	2.000	1.962	0.683	0.490	0.497
**Locus 20**	0.081	0.041	0.959	37.000	2.000	1.086	0.172	0.079	0.080
**Locus 21**	0.919	0.715	0.285	37.000	2.000	1.687	0.597	0.407	0.413
**Locus 22**	0.081	0.041	0.959	37.000	2.000	1.086	0.172	0.079	0.080
**Locus 23**	0.459	0.265	0.735	37.000	2.000	1.638	0.578	0.389	0.395
**Locus 24**	0.459	0.265	0.735	37.000	2.000	1.638	0.578	0.389	0.395
**Mean**				37	2	1.448	0.436	0.280	0.284
