# Supplementary material for: Development and use of miRNA-derived SSR markers for the study of genetic diversity, population structure, and characterization of genotypes for breeding heat tolerant wheat varieties
Source: PLoS One. 2021 Feb 4;16(2):e0231063. doi: 10.1371/journal.pone.0231063 (PMC7861453; doi:10.1371/journal.pone.0231063)
Supplement: S4 Table — (PDF) [file pone.0231063.s005.pdf]

Supplementary Table 4: Genetic variation of individual locus in four populations including the sample size, number of different alleles (Na), the number of effective allele (Ne), Shannon's index (I), expected heterozygosity (He) and unbiased expected heterozygosity (uHe).

Pop	Locus	Band Freq.	p	q	N	Na	Ne	I	He	uHe
**Pop1**	**Locus 1**	0.417	0.236	0.764	12.000	2.000	1.565	0.547	0.361	0.377
	**Locus 2**	0.583	0.355	0.645	12.000	2.000	1.844	0.650	0.458	0.478
	**Locus 3**	0.250	0.134	0.866	12.000	2.000	1.302	0.394	0.232	0.242
	**Locus 4**	0.750	0.500	0.500	12.000	2.000	2.000	0.693	0.500	0.522
	**Locus 5**	0.417	0.236	0.764	12.000	2.000	1.565	0.547	0.361	0.377
	**Locus 6**	0.250	0.134	0.866	12.000	2.000	1.302	0.394	0.232	0.242
	**Locus 7**	0.333	0.184	0.816	12.000	2.000	1.428	0.477	0.300	0.313
	**Locus 8**	0.083	0.043	0.957	12.000	2.000	1.089	0.176	0.082	0.085
	**Locus 9**	0.000	0.000	1.000	12.000	0.000	1.000	0.000	0.000	0.000
	**Locus 10**	0.750	0.500	0.500	12.000	2.000	2.000	0.693	0.500	0.522
	**Locus 11**	0.167	0.087	0.913	12.000	2.000	1.189	0.296	0.159	0.166
	**Locus 12**	0.083	0.043	0.957	12.000	2.000	1.089	0.176	0.082	0.085
	**Locus 13**	0.167	0.087	0.913	12.000	2.000	1.189	0.296	0.159	0.166
	**Locus 14**	0.250	0.134	0.866	12.000	2.000	1.302	0.394	0.232	0.242
	**Locus 15**	0.083	0.043	0.957	12.000	2.000	1.089	0.176	0.082	0.085
	**Locus 16**	0.500	0.293	0.707	12.000	2.000	1.707	0.605	0.414	0.432
	**Locus 17**	0.500	0.293	0.707	12.000	2.000	1.707	0.605	0.414	0.432
	**Locus 18**	0.583	0.355	0.645	12.000	2.000	1.844	0.650	0.458	0.478
	**Locus 19**	0.417	0.236	0.764	12.000	2.000	1.565	0.547	0.361	0.377
	**Locus 20**	0.083	0.043	0.957	12.000	2.000	1.089	0.176	0.082	0.085
	**Locus 21**	0.917	0.711	0.289	12.000	2.000	1.697	0.601	0.411	0.429
	**Locus 22**	0.250	0.134	0.866	12.000	2.000	1.302	0.394	0.232	0.242
	**Locus 23**	0.583	0.355	0.645	12.000	2.000	1.844	0.650	0.458	0.478
	**Locus 24**	0.167	0.087	0.913	12.000	2.000	1.189	0.296	0.159	0.166
**Pop2**	**Locus 1**	0.400	0.225	0.775	10.000	2.000	1.537	0.534	0.349	0.368
	**Locus 2**	0.600	0.368	0.632	10.000	2.000	1.869	0.658	0.465	0.489
	**Locus 3**	0.100	0.051	0.949	10.000	2.000	1.108	0.202	0.097	0.102
	**Locus 4**	0.500	0.293	0.707	10.000	2.000	1.707	0.605	0.414	0.436
	**Locus 5**	0.900	0.684	0.316	10.000	2.000	1.762	0.624	0.432	0.455
	**Locus 6**	0.100	0.051	0.949	10.000	2.000	1.108	0.202	0.097	0.102
	**Locus 7**	0.000	0.000	1.000	10.000	0.000	1.000	0.000	0.000	0.000
	**Locus 8**	0.000	0.000	1.000	10.000	0.000	1.000	0.000	0.000	0.000
	**Locus 9**	0.500	0.293	0.707	10.000	2.000	1.707	0.605	0.414	0.436
	**Locus 10**	0.300	0.163	0.837	10.000	2.000	1.376	0.445	0.273	0.288
	**Locus 11**	0.200	0.106	0.894	10.000	2.000	1.233	0.337	0.189	0.199
	**Locus 12**	0.000	0.000	1.000	10.000	0.000	1.000	0.000	0.000	0.000
	**Locus 13**	0.500	0.293	0.707	10.000	2.000	1.707	0.605	0.414	0.436
	**Locus 14**	0.300	0.163	0.837	10.000	2.000	1.376	0.445	0.273	0.288

	**Locus 15**	0.200	0.106	0.894	10.000	2.000	1.233	0.337	0.189	0.199
	**Locus 16**	0.000	0.000	1.000	10.000	0.000	1.000	0.000	0.000	0.000
	**Locus 17**	0.700	0.452	0.548	10.000	2.000	1.982	0.689	0.495	0.522
	**Locus 18**	0.200	0.106	0.894	10.000	2.000	1.233	0.337	0.189	0.199
	**Locus 19**	0.800	0.553	0.447	10.000	2.000	1.978	0.688	0.494	0.520
	**Locus 20**	0.200	0.106	0.894	10.000	2.000	1.233	0.337	0.189	0.199
	**Locus 21**	0.800	0.553	0.447	10.000	2.000	1.978	0.688	0.494	0.520
	**Locus 22**	0.000	0.000	1.000	10.000	0.000	1.000	0.000	0.000	0.000
	**Locus 23**	0.300	0.163	0.837	10.000	2.000	1.376	0.445	0.273	0.288
	**Locus 24**	0.700	0.452	0.548	10.000	2.000	1.982	0.689	0.495	0.522
**Pop3**	**Locus 1**	0.250	0.134	0.866	8.000	2.000	1.302	0.394	0.232	0.248
	**Locus 2**	0.750	0.500	0.500	8.000	2.000	2.000	0.693	0.500	0.533
	**Locus 3**	0.375	0.209	0.791	8.000	2.000	1.495	0.513	0.331	0.353
	**Locus 4**	0.625	0.388	0.612	8.000	2.000	1.904	0.668	0.475	0.506
	**Locus 5**	0.625	0.388	0.612	8.000	2.000	1.904	0.668	0.475	0.506
	**Locus 6**	0.125	0.065	0.935	8.000	2.000	1.137	0.239	0.121	0.129
	**Locus 7**	0.125	0.065	0.935	8.000	2.000	1.137	0.239	0.121	0.129
	**Locus 8**	0.000	0.000	1.000	8.000	0.000	1.000	0.000	0.000	0.000
	**Locus 9**	0.125	0.065	0.935	8.000	2.000	1.137	0.239	0.121	0.129
	**Locus 10**	0.625	0.388	0.612	8.000	2.000	1.904	0.668	0.475	0.506
	**Locus 11**	0.250	0.134	0.866	8.000	2.000	1.302	0.394	0.232	0.248
	**Locus 12**	0.000	0.000	1.000	8.000	0.000	1.000	0.000	0.000	0.000
	**Locus 13**	0.000	0.000	1.000	8.000	0.000	1.000	0.000	0.000	0.000
	**Locus 14**	0.250	0.134	0.866	8.000	2.000	1.302	0.394	0.232	0.248
	**Locus 15**	0.375	0.209	0.791	8.000	2.000	1.495	0.513	0.331	0.353
	**Locus 16**	0.375	0.209	0.791	8.000	2.000	1.495	0.513	0.331	0.353
	**Locus 17**	0.500	0.293	0.707	8.000	2.000	1.707	0.605	0.414	0.442
	**Locus 18**	0.250	0.134	0.866	8.000	2.000	1.302	0.394	0.232	0.248
	**Locus 19**	0.750	0.500	0.500	8.000	2.000	2.000	0.693	0.500	0.533
	**Locus 20**	0.000	0.000	1.000	8.000	0.000	1.000	0.000	0.000	0.000
	**Locus 22**	0.000	0.000	1.000	8.000	0.000	1.000	0.000	0.000	0.000
	**Locus 23**	0.500	0.293	0.707	8.000	2.000	1.707	0.605	0.414	0.442
	**Locus 24**	0.500	0.293	0.707	8.000	2.000	1.707	0.605	0.414	0.442
**Pop4**	**Locus 1**	0.571	0.345	0.655	7.000	2.000	1.825	0.645	0.452	0.487
	**Locus 2**	0.429	0.244	0.756	7.000	2.000	1.585	0.556	0.369	0.397
	**Locus 3**	0.429	0.244	0.756	7.000	2.000	1.585	0.556	0.369	0.397
	**Locus 4**	0.429	0.244	0.756	7.000	2.000	1.585	0.556	0.369	0.397
	**Locus 5**	0.429	0.244	0.756	7.000	2.000	1.585	0.556	0.369	0.397
	**Locus 6**	0.000	0.000	1.000	7.000	0.000	1.000	0.000	0.000	0.000
	**Locus 7**	0.286	0.155	0.845	7.000	2.000	1.355	0.431	0.262	0.282

	**Locus 8**	0.000	0.000	1.000	7.000	0.000	1.000	0.000	0.000	0.000
	**Locus 9**	0.429	0.244	0.756	7.000	2.000	1.585	0.556	0.369	0.397
	**Locus 10**	0.286	0.155	0.845	7.000	2.000	1.355	0.431	0.262	0.282
	**Locus 11**	0.286	0.155	0.845	7.000	2.000	1.355	0.431	0.262	0.282
	**Locus 12**	0.000	0.000	1.000	7.000	0.000	1.000	0.000	0.000	0.000
	**Locus 13**	0.143	0.074	0.926	7.000	2.000	1.159	0.264	0.137	0.148
	**Locus 14**	0.429	0.244	0.756	7.000	2.000	1.585	0.556	0.369	0.397
	**Locus 15**	0.143	0.074	0.926	7.000	2.000	1.159	0.264	0.137	0.148
	**Locus 16**	0.286	0.155	0.845	7.000	2.000	1.355	0.431	0.262	0.282
	**Locus 17**	0.714	0.465	0.535	7.000	2.000	1.991	0.691	0.498	0.536
	**Locus 18**	0.143	0.074	0.926	7.000	2.000	1.159	0.264	0.137	0.148
	**Locus 19**	0.857	0.622	0.378	7.000	2.000	1.888	0.663	0.470	0.506
	**Locus 20**	0.000	0.000	1.000	7.000	0.000	1.000	0.000	0.000	0.000
	**Locus 22**	0.000	0.000	1.000	7.000	0.000	1.000	0.000	0.000	0.000
	**Locus 23**	0.429	0.244	0.756	7.000	2.000	1.585	0.556	0.369	0.397
	**Locus 24**	0.571	0.345	0.655	7.000	2.000	1.825	0.645	0.452	0.487
